# Femtosecond Laser Ablation and Delamination of Functional Magnetic Multilayers at the Nanoscale

**DOI:** 10.3390/nano14181488

**Published:** 2024-09-13

**Authors:** Pavel Varlamov, Jan Marx, Yoav Urbina Elgueta, Andreas Ostendorf, Ji-Wan Kim, Paolo Vavassori, Vasily Temnov

**Affiliations:** 1 LSI, Ecole Polytechnique, CEA/DRF/IRAMIS, CNRS, Institut Polytechnique de Paris, 91128 Palaiseau, France; pavel.varlamov@polytechnique.edu; 2 Applied Laser Technologies, Ruhr University Bochum, Universitätsstraße 150, 44801 Bochum, Germany; jan.marx@ruhr-uni-bochum.de (J.M.);; 3 CIC nanoGUNE—BRTA, Donostia—San Sebastian, 20018 Donostia-San Sebastian, Spain; 4 Department of Polymers and Advanced Materials: Physics, Chemistry and Technology, Faculty of Chemistry, University of the Basque Country UPV/EHU, 20018 Donostia-San Sebastián, Spain; 5 Department of Physics, Kunsan National University, Kunsan 54150, Republic of Korea; 6 IKERBASQUE, Basque Foundation for Science, 48009 Bilbao, Spain

**Keywords:** laser ablation, laser delamination, fs-laser pulses, thresholds, Ni thin films, Ni/Au bilayers, heat penetration depth

## Abstract

Laser nanostructuring of thin films with ultrashort laser pulses is widely used for nanofabrication across various fields. A crucial parameter for optimizing and understanding the processes underlying laser processing is the absorbed laser fluence, which is essential for all damage phenomena such as melting, ablation, spallation, and delamination. While threshold fluences have been extensively studied for single compound thin films, advancements in ultrafast acoustics, magneto-acoustics, and acousto-magneto-plasmonics necessitate understanding the laser nanofabrication processes for functional multilayer films. In this work, we investigated the thickness dependence of ablation and delamination thresholds in Ni/Au bilayers by varying the thickness of the Ni layer. The results were compared with experimental data on Ni thin films. Additionally, we performed femtosecond time-resolved pump-probe measurements of transient reflectivity in Ni to determine the heat penetration depth and evaluate the melting threshold. Delamination thresholds for Ni films were found to exceed the surface melting threshold suggesting the thermal mechanism in a liquid phase. Damage thresholds for Ni/Au bilayers were found to be significantly lower than those for Ni and fingerprint the non-thermal mechanism without Ni melting, which we attribute to the much weaker mechanical adhesion at the Au/glass interface. This finding suggests the potential of femtosecond laser delamination for nondestructive, energy-efficient nanostructuring, enabling the creation of high-quality acoustic resonators and other functional nanostructures for applications in nanosciences.

## 1. Introduction

Ultrashort laser pulses provide significant potential for the precise and efficient fabrication of nano- and microstructures on metal surfaces. These structures can impart surfaces with unique properties, including antireflective characteristics [1,2,3], enhanced plasmonic effects [4,5], antibacterial effects [6,7], and modified wettability [8,9].

Ablation is still one of the most common phenomena to produce nanostructures. Experiments have demonstrated that ultrashort irradiation allows for better control and precision in material modification compared to longer pulses [10,11]. However, laser ablation occurs as a result of laser-induced phase transitions, involving a series of highly nonequilibrium states of matter that are difficult to control [12,13,14]. Femtosecond laser ablation typically requires laser-induced melting, which triggers the complex spatiotemporal hydrodynamic/mechanical/acoustic motion in the melted material [15,16]. The resulting resolidified surface modifications can unpredictably alter material properties, which limits this approach for the applications.

Another promising method for producing structures is laser spallation. This phenomenon involves the delamination or separation of the film from the film-substrate boundary due to the transient tensile stress generated by the laser pulse. Laser spallation has been known since the 1970s, when it was first observed in experiments involving the irradiation of macroscopically thick metallic films with nanosecond pulses [17,18]. In the late 1990s and early 2000s, the phenomenon was demonstrated using femtosecond laser pulses, albeit still on microscopically thick films [19]. In 2004, thermoelastic ‘jet formation of nanobumps’ was demonstrated on 60 nm gold films [20]. Domke et al. referred to this process as ‘bulging’ in their investigations of damage mechanisms in Mo thin films of varying thicknesses [21]. Both aforementioned studies evidence the controlled separation of the film from the substrate without creating a hole, to be denoted as delamination throughout this paper. In 2020, the creation of closed cavities through femtosecond laser-induced internal thermo-mechanical laser spallation inside 300 nm Ni films has been discovered [22]. Fabrication of closed cavities in Ni thin films and their periodic arrangements is crucial for studying phonon–magnon interactions [23,24].

The key parameter for optimizing the laser nanofabrication process and understanding the underlying damage phenomena is the fluence threshold. This threshold defines the minimum amount of deposited laser energy required to initiate ablation/spallation/delamination processes. Researchers widely investigate this parameter to comprehend the processes to optimize nanofabrication [10,21,25,26,27].

However, research interest also extends to the use of more complex multilayered films. Multilayers are of significant interest in fields such as ultrafast magneto-plasmonics [28,29], ultrafast acoustics [30,31], ultrafast magneto-acoustics [32,33], and ultrafast acousto-magneto-plasmonics [34]. These applications require the use of laser pulses for the nanostructuring of multilayered films, while mostly fundamental aspects of light-matter interaction are considered in mono compound thin films. Although there are studies on multilayer films, most are theoretical [35,36] or consider a single configuration without varying the thickness of the consistent layers [37,38]. In [39], the authors investigated the damage mechanism in Au/Ti bilayers by varying the thickness of the Au layer. They found that increasing the thickness of Au reduces the rates of melting and evaporation but enhances the mechanical response. However, threshold fluences were not provided.

In addition, theoretical investigations of phonon–magnon coupling in free-standing Ni/Au bilayers and Au/Ni/Au structures have revealed the potential to generate high-order magnon modes with frequencies exceeding 100 GHz [40]. In these structures, the magnon spectrum is solely determined by the thickness of the ferromagnetic layer, while the spectrum of acoustic vibrations depends on the total thickness of the multilayer. This allows for independent control and frequency/phase matching between individual phonon and magnon modes. The production of these structures is highly desirable, and delamination can be employed for this purpose.

In this article, we make a step forward in understanding the laser nanofabrication of complex magnetic multilayers. We investigated the damage thresholds of bilayer Ni/Au and Ni thin films, focusing on the influence of the Ni layer thickness. Experiments were conducted using single-pulse laser irradiation to determine ablation and spallation/delamination thresholds, which were then compared with the estimated melting thresholds. To achieve this, the heat penetration depth was derived from an analysis of pump-probe measurements of transient reflectivity. The analysis confirmed the thermal nature of both ablation and delamination, though the thresholds for Ni/Au films were significantly lower than those for Ni films.

## 2. Materials and Methods

### 2.1. Experimental Details: Laser Irradiation

We conducted a series of single-shot experiments using a conventional setup on Ni/Au bilayers, where a 5 nm Au layer was sandwiched between the glass substrate and Ni films of varying thicknesses (10–150 nm). Additionally, we examined Ni films of varying thicknesses (20–150 nm) deposited on glass substrates. All samples were made by DC sputtering deposition on a Borofloat33 substrate of dimension 10 × 10 × 0.65 mm, base pressure of 1 × 10^−8^ Torr, and deposition pressure of 3 × 10^−3^ Torr, with a rotation of the sample of 20 rpm. The rate of deposition for Au is 7.9 nm/min with a power of 50 Watts, i.e., was deposited for 38 sec to obtain 5 nm; on the other hand, for Ni, a power of 150 Watts was used to obtain a deposition rate of 7.8 nm/min for the sample with gold and 7.5 nm/min for the sample without gold (the difference between this deposition rate was due to the environment of the sputtering chamber and the difference in calibration in these new conditions).

The films were irradiated using a Spitfire Spectra-Physics laser delivering 110 fs pulses at an 800 nm wavelength in a single-shot regime, under two configurations: from the air side and the substrate side, with the s-polarized beam incident on the sample at a 45° angle. In both cases, the irradiation was performed in the atmospheric air. The collimated beam was focused using a lens with a focal length of 40 cm. The pulse energy was varied up to the maximum available value of 16.7 µJ.

The ablation thresholds were determined using Liu’s method by measuring the lateral dimensions of craters at different fluences from microphotographs [41]. The fluence distribution *F(r)* follows a Gaussian distribution:

(1) $$F(r)=F_{p}exp\left(-\frac{r^{2}}{a^{2}}\right),$$

where *a* is a spot size and *F_p_* is the pulse fluence, which can be expressed in terms of the pulse energy as *E_p_ = πa*^2^*F_p_*. From Equation (1), the relationship between the crater size and the pulse energy can be derived as

(2) $$r^{2}=a^{2}ln\left(\frac{E_{p}}{\pi a^{2}F(r)}\right).$$

Plotting the square of the structure radius against the logarithm of energy should yield a linear relationship. The line intersects at *r =* 0 for *F(r) = F_th_*, thereby determining the threshold energy. The beam size, *a*, corresponds to the slope of the line. Since the beam was focused at a 45° angle in the experiments, both the beam shape and the resulting structures were elliptical. Applying the previous considerations based on Liu’s method for two radii, we determined the beam spot size to be approximately 30 × 40 µm, as indicated by the intercept of the slope described in Equation (2).

The value of *E_p_* here represents the energy pulse incident on the sample. To determine the fluence absorbed by the sample, we accounted for the reflectivity and transmission coefficients. For air side irradiation, the absorbed threshold was calculated as *F_abs_ = AF_th_,* where *A* = (1 − *R* − *T),* with *R* and *T* being the reflectivity and transmittance, respectively. In the case of glass side irradiation, the reflection from the glass side was also considered, resulting in *F_abs_ = T_glass_AF_th_.* The reflectivity and transmittance values for Ni and Ni/Au of different thicknesses were calculated for s-polarized light using the transfer matrix approach [42,43]. The values of the used coefficients are provided in Table 1. *T_glass_* was taken to be 0.9. Taking the reflectivity and transmission coefficients into account allows us to eliminate their impact on the threshold values.

### 2.2. Estimation of Melting Thresholds

To estimate the damage thresholds, we followed a similar approach as in [21]. The idea is to estimate the threshold fluences required for melting. These calculations were then compared with the experimentally obtained values of the ablation and delamination threshold. A specific energy density E/V is necessary to initiate melting, which is given by

(3) $$\frac{E_{m}}{V}=\rho (c_{l}(T_{m}-T_{0})+\Delta H_{m}),$$

where *ρ* is the mass density; *c_l_* is the heat capacity; *T_m_* and *T*_0_ are the melting and room temperature, respectively; and ∆*H_m_* is the melting heat. The values of these parameters are listed in Table 2.

Assuming that the energy density decreases exponentially with the depth of the material, the threshold fluence for melting can be expressed as

(4) $$F_{m}=\int_{0}^{d}\frac{E_{m}}{V}e^{-z/h}dz,$$

where *h* is the heat penetration depth describing how deeply heat penetrates into the material. Integrating from 0 to *d*, the thickness of the film yields

(5) $$F_{m}=\rho (c_{l}(T_{m}-T_{0})+dH_{m})h(1-e^{-d/h}).$$

The heat penetration depth is a key parameter for estimating the melting threshold. In 1988, Corkum et al. derived this parameter as a function of the two-temperature model coefficients [45]:

(6) $$h={\left(\frac{128}{\pi }\right)}^{1/8}{\left(\frac{K_{0}^{2}C_{l}}{T_{m}G^{2}\gamma }\right)}^{1/4},$$

where *K*_0_ is the thermal conductivity, *C_l_* is the lattice heat capacity, *G* is the electron–phonon coupling constant, and *γ* is the electron heat capacity.

Unlike the electron–phonon coupling factor *G* and the other parameters, the heat penetration depth h can be more straightforwardly obtained from experiments. This is typically determined through pump-probe measurements of transient reflectivity, which changes upon the generation of acoustic pulses induced by laser irradiation. By analyzing these reflectivity changes, the heat penetration depth can be evaluated. To check the intensity dependence of the heat penetration depth, pump intensity-dependent measurements were performed. A detailed analysis is provided in Section 3.2 of the Results section, while the next subsection outlines the experimental details of the pump-probe measurements.

### 2.3. Experimental Details: Pump-Probe Measurements of Transient Reflectivity

To determine the heat penetration depth required for estimating the melting thresholds of Ni, pump-probe measurements of transient reflectivity were conducted. The pump pulse, at 400 nm and a repetition rate of 1 kHz, was applied from both the air side and the substrate side of a 240 nm Ni film. The pump was focused to a spot size of 300 µm. These measurements were performed on Ni films deposited on sapphire and glass substrates. The probe pulse, at 800 nm, was applied from the air side of the sample and focused to a spot size of 80 µm. Both the pump and probe pulses had durations of 60 fs. Measurements were taken at various pump pulse intensities to examine the dependence of the heat penetration depth on intensity.

## 3. Results

### 3.1. Results on the Irradiation of Ni and Ni/Au Films

**Figure 1:**
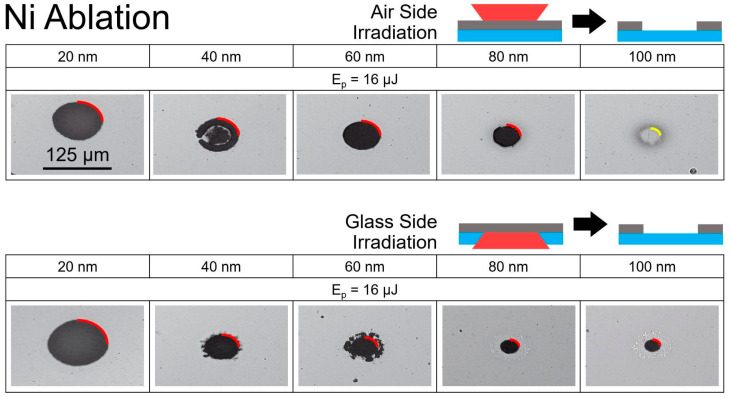
Optical microscopy of the structures obtained by a single fs-laser pulse on the Ni films in reflection. The upper set of photos demonstrates the structures for the different thicknesses of Ni film produced by the laser pulse coming from air at the same pulse energy (E_p_ = 16 μJ). The lower set shows the same for the case of irradiation from the substrate side. The red quarter circles indicate the diameters of ablation craters, where the film was completely removed. The yellow quarter circle for air side ablation of 100 nm Ni indicates the ablation crater due to surface ablation. The scale, indicated for the 20 nm Ni ablation in air at 125 μm, applies to all other micrographs.

The results of laser irradiation on Ni films from both the air side and the glass side of the sample are depicted in Figure 1. A noticeable decrease in the lateral size of the craters for different film thicknesses, obtained at a pulse energy of 16 μJ, was evident in both cases. All the craters, with the exception of the 100 nm Ni case, appeared significantly darker than the original films (Figure 1). This darkness indicates the complete removal of the film, as the dark craters revealed the underlying glass surface, which had a much lower reflectivity than Ni (4% for glass versus 66% for Ni). This difference in reflectivity caused the craters to appear dark in reflected light microscopy images. Ablation is defined as the complete removal of the film from the surface, producing well-defined holes in the film. Hole sizes were determined based on optical microscopy marked by red quarter-circular lines along their edges (see Figure 1). For the 100 nm Ni film, the laser intensity from the air side was insufficient to ablate the entire film but resulted in the removal of a few nanometers of Ni from the Ni–air interface forming a shallow ablation crater. This case, which we denote as surface ablation, is the common case for femtosecond laser ablation of macroscopically thick, semi-infinite solid targets. For the surface ablation, the boundary of the crater is marked by a yellow quarter-circular line in Figure 1.

**Figure 2:**
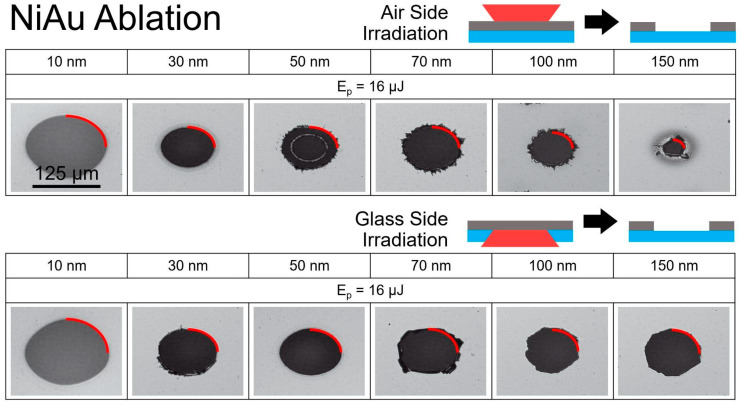
Optical microscopy of the structures obtained by a single fs-laser pulse on the Ni/Au films in reflection. The upper set of photos demonstrates the structures for the different thicknesses of Ni/Au film produced by the laser pulse coming from air at a specified energy pulse (E_p_ = 16 μJ). The lower panel shows the same for the case of irradiation from the substrate side. The red quarter circles indicate the diameters of ablation holes, where the film was completely removed (ablated). The thickness mentioned for NiAu refers to the thickness of the Ni layer deposited on a 5 nm Au layer, which is situated on the glass substrate. The scale, indicated for the 10 nm NiAu ablation in air at 125 μm, applies to all other micrographs.

Figure 2 illustrates the results of irradiating Ni/Au films. The dimensions of ablation holes were measured along the red quarter circles. Generally, the holes are larger for Ni/Au compared to Ni films of similar thicknesses under the same irradiation conditions. A significant size difference was observed for the 10 nm and 150 nm Ni/Au films in the case of air-side ablation. For thicknesses from 30 to 100 nm, the visible change was less pronounced. In the case of glass-side irradiation, a noticeable change was observed only for the 10 nm film, while the lateral sizes of the holes for other thicknesses remained consistent.

**Figure 3:**
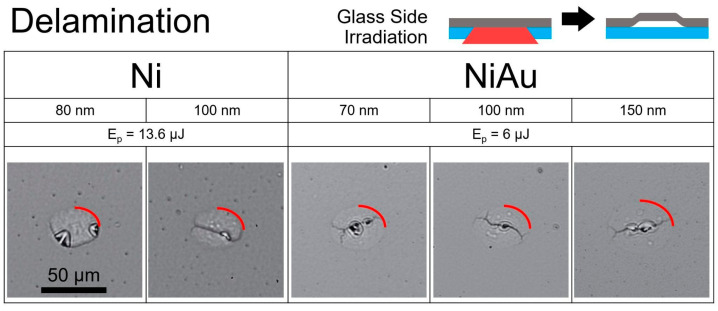
Optical microscopy of the structures obtained by a single fs-laser pulse on Ni and Ni/Au films on glass in reflection. The set of photos demonstrates the structures for the different thicknesses of Ni and Ni/Au film produced by the laser pulse coming from the glass side at specified energy pulses. The red quarter circles indicate the diameters of structures, obtained with delamination. The thickness mentioned for NiAu refers to the thickness of the Ni layer deposited on a 5 nm Au layer, which was situated on the glass substrate. The scale, indicated for the 80 nm Ni delamination at 50 μm, applies to all other micrographs.

Irradiation below the ablation thresholds from the glass side of the samples led to the delamination of the films. The resulting structures are shown in Figure 3. For Ni films, delamination was observed for thicknesses of 80 nm and 100 nm with pulse energies below 14 μJ. The delamination structures on Ni/Au bilayers were obtained with lower pulse energies (Ep < 7 μJ) for Ni thicknesses of 70 nm, 100 nm, and 150 nm.

**Figure 4:**
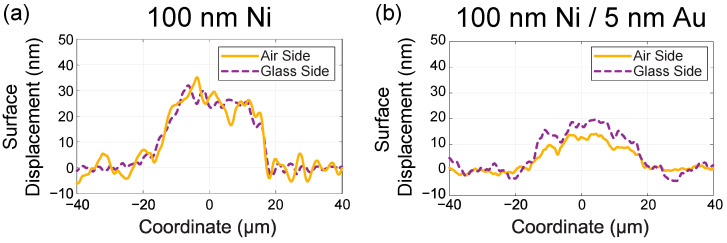
Surface displacement of Ni (**a**) and Ni/Au (**b**) delaminated films on both air and glass sides, as measured by interferometry.

While the complete removal of the films is clearly indicated by the formation of dark holes following laser irradiation, the delamination process is less apparent. To examine the morphology of the resulting structures, interferometry measurements were conducted. Interferometry, a non-contact method for analyzing surface morphology, enables the examination of both the top surface and the profile of the glass-side surface. We utilized Michelson interferometry in the Linnik configuration, employing quantitative Fourier algorithms for profile reconstruction [22]. The results reveal a surface displacement of 20–30 nm for the 100 nm Ni film from both sides (Figure 4a). A smaller displacement of 10–20 nm was observed for the Ni(100 nm)/Au(5 nm) bilayer (Figure 4b). These findings indicate the presence of delamination. Films with thinner layers show smaller or similar displacements.

### 3.2. Evaluation of the Heat Penetration Depth from the Analysis of Pump-Probe Measurements

**Figure 5:**
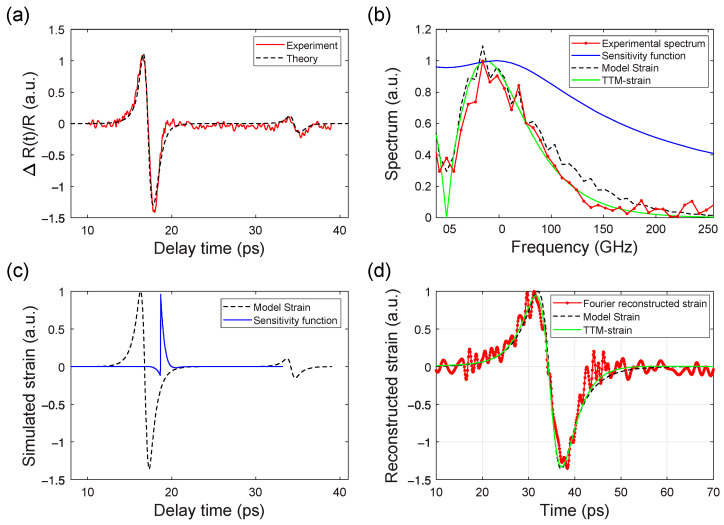
(**a**) Time-resolved reflectivity measurements of acoustic pulses in a Ni (240 nm)/sapphire sample, obtained by pumping at the nickel–air interface and probing at the nickel–sapphire interface, alongside the modeled reflectivity. (**b**) Acoustic spectrum derived from the experimental reflectivity data and the sensitivity function used for modeling the acoustic spectrum. (**c**) Simulated acoustic pulse and the sensitivity function employed in the modeling process. (**d**) Reconstructed strain obtained from experimental data and the corresponding modeled strain. The reflectivity data were well-approximated using a model strain with an exponential decay characterized by a heating depth *h* = 24 nm. Both the acoustic spectra and pulses were well-reproduced using the two-temperature (TTM) model by Saito et al. [44]. The Fourier reconstructed strain was derived using the algorithm developed by Manke et al. [30]. Time-resolved reflectivity measurements enable precise evaluation of the heating depth *h* up to the damage threshold.

The key parameter in destructive laser interactions with materials is the energy deposition (or heat penetration) depth *h*. One of the most direct ways to evaluate relies on picosecond laser ultrasonics [46]. The shape of ultrashort acoustic pulses generated by the thermal expansion of a then near-surface layer of laser-heated material mimics the initial heat profile. However, the extraction of the heat penetration depth is a delicate issue as it relies on an interplay of usually unknown photo-elastic coefficients, which depend on the material and probe wavelength [47]. Fourier-based methods of strain reconstruction Picosecond acoustic phonon pulse generation in nickel and chromium [30,48] are usually sensitive to photoelastic coefficients, notably for common detection schemas of acoustic pulses during their reflection from a free interface. We analyzed experimental configurations and found that acoustic pulses can be best quantified while traversing an interface with an optically transparent and acoustically matched substrate. An example of such measurement is presented in Figure 5. The dynamics of optical reflectivity measured at the Ni(240 nm)/sapphire interface evidence the generation and propagation of ultrashort acoustic pulses generated at the nickel-air interface (Figure 5a). Approximately 40 ps after laser excitation, the acoustic pulse arrived at the nickel–sapphire interface and induced a strong variation of the refractive index upon injection in the sapphire substrate. A much smaller acoustic echo observed at 120 ps evidenced a small (~10%) acoustic reflection from the nickel–sapphire interface. After some simple math [30], the time-resolved reflectivity signal in Figure 5a can be represented as a convolution of the acoustic strain pulse with the optical sensitivity function in the frequency (Figure 5b) and time domain (Figure 5c). A model strain pulse consisting of two exponentially decaying tails ~exp(−|t − t_0_|/τ) with τ = 4 ps provides an accurate fit to experimental data, shown in Figure 5d. The heat penetration depth was extracted as τ × ʋ = 24 nm. This conclusion is reinforced by more detailed simulations based on the two-temperature model (TTM) accounting for ultrafast optical excitation of nonequilibrium hot electrons in nickel, their diffusion, and cooling down due to interaction with cold phonons. Using the set of parameters and analytical equations by Saito and co-workers [48], we were able to reproduce the model strain from the TTM-model by adjusting the only parameter, i.e., the temperature of the film to be 300 degrees Celsius. This temperature represents a reasonable value for a nickel thin film on a dielectric substrate excited by a train of femtosecond laser pulses at a repetition rate of 1 kHz. It should be noted that the presented measurements were performed 10% below the multipulse damage threshold of the investigated sample. Performing simulations with arbitrary photoelastic coefficients, we confirmed that the extracted heat penetration depth can be seen as a reliable fitting parameter with a 20% error bar, i.e., 20 nm < *h* < 30 nm.

Substituting the parameters from Saito and co-workers in Equation (6) gives the value *h* = 28 nm. Therefore, we conclude that our measurements of the heat penetration depth *h* coincide very well with previously reported results.

While searching for intensity-dependent effects, we performed intensity-dependent photoacoustic measurements by increasing the pump intensity over two orders of magnitude until reaching the multipulse damage threshold. The shape of acoustic echoes remained identical. This observation is in line with the theoretical predictions of Milov and Medvedev [49] who report on pump-independent electron–phonon coupling constants in Ni. At the same time, it challenges the Lin and Zhigilei [50] study anticipating the strong intensity dependence of electron–phonon coupling in nickel. Irrespective of poorly understood physics behind femtosecond laser interactions with materials in the destructive regime, the presented way to extract the heat penetration depth allows for an unambiguous interpretation of the reported damage thresholds based on the energy conservation law as quantified by Equation (5).

## 4. Discussion

**Figure 6:**
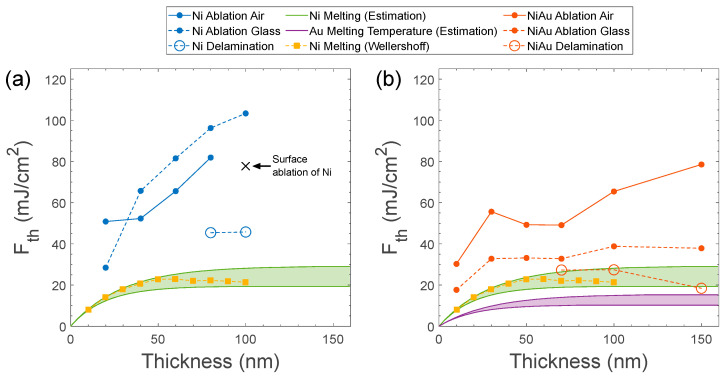
Ablation and delamination thresholds as a function of thickness for Ni (**a**) and Ni/Au (**b**) thin films, subjected to irradiation from both the air and substrate sides. The individual dots denote the thresholds evaluated using Liu’s method (for ease of visual interpretation, these dots are interconnected by lines). The black crossed point in (**a**) is a threshold value for incomplete Ni ablation. The green lines and the shaded area represent the calculated nickel/substrate interface melting threshold given by Equation (5) for *h* in a range from 20 nm to 30 nm. The purple lines/shaded area represent the calculated fluence required to heat the nickel at the Ni/Au interface to the melting temperature of gold (1377 K). Yellow squares represent the fs-laser-induced melting thresholds at the nickel–air surface, experimentally obtained by Wellershoff et al. [26]. The thickness mentioned for NiAu refers to the thickness of the Ni layer deposited on a 5 nm Au layer, which is situated on the glass substrate.

The evaluated thresholds are summarized in Figure 6. In order to interpret these thresholds, it makes sense to reference them to the melting threshold in fs-laser-excited Ni. The latter was obtained in two distinct ways. First, we used the experimental data by Wellershoff et al. [28]. Second, we calculated the melting threshold using Equation (5) and the heat penetration depth *h =* 25 nm obtained from the time-resolved reflectivity measurements. Quantitative agreement between these two curves in Figure 6a justifies the accuracy of both methods. As such, all damage thresholds in Figure 6a were found to exceed the surface/interface melting threshold in Ni. Therefore, we conclude that in the case of glass side excitation, nickel undoubtedly melts at the glass–nickel interface. Both the ablation and delamination mechanism would be mediated by the disruption of liquid nickel by giant negative pressures of the order of −5…−10 GPa generated by the complex thermo-elastic dynamics in partially melted nickel layers [23]. The amount of the absorbed laser fluence is definitely not enough to melt the entire nickel layer, suggesting that nickel always remains in the solid state at a Ni/air interface. For 80 nm and 100 nm nickel, thin film ablation occurs via the delamination of the entire film that tends to form closed cavities. The ablation mechanism is due to the disruption of the delaminated film by lateral stresses.

The situation is quite different for the ablation from the Ni/air interface, which requires much more energy for the material to be removed. Let us take a closer look at the ablation threshold of a 100 nm thin film, where 100 mJ/cm^2^ is needed to ablate the entire film. That threshold is higher than the surface ablation threshold of Ni (80 mJ/cm^2^, cross symbol in Figure 6a), resulting in a removal of a few nanometers of nickel from the Ni/air interface evidenced by optical interference microscopy. If we assume that the absorbed laser energy of 100 mJ/cm^2^ is homogeneously distributed over the entire nickel due to thermal diffusion, we obtain higher fluence than the melting threshold given by Equation (5) (=88 mJ/cm^2^ for *h >> d*). Therefore, we cannot rule out that the air-side ablation of 100 nm nickel films is due to the melting of the entire film.

The analysis based on the energy conservation law results in a different interpretation of damage thresholds for Ni/Au films in Figure 6b. For both glass-side and air-side irradiation, the damage thresholds for Ni/Au bilayers were lower than those for Ni thin films. This trend was more pronounced for thicker films, while for thinner films, it remained evident but less distinct. The difference in thresholds clarify why the crater diameters were larger for Ni/Au films compared to Ni films (Figure 1, Figure 2 and Figure 3).

Let us discuss the air-side ablation first. Being significantly higher than the calculated melting threshold at the Ni/air interface (=25 mJ/cm^2^), the measured air-side ablation threshold fluence (=80 mJ/cm^2^ for Ni(150 nm)/Au(5 nm) bilayer, see Figure 6b) was still not sufficient to achieve the transient melting of the entire nickel film (=130 mJ/cm^2^). With the same argument, unfortunately, we cannot rule out that the 5 nm thin gold layer gets melted. An assumption that gold melts when the adjacent nickel layer is homogeneously heated to the melting temperature of gold (1337 K, which is below the melting temperature of Ni [26]) would require the minimum absorbed laser fluence to be 70 mJ/cm^2^, which is slightly below the ablation threshold. Therefore, we conclude that the gold layer could melt and the mechanism of the air-side ablation of Ni(150 nm)/Au(5 nm) bilayers is of non-thermal origin only in a sense that Ni does not melt.

The same analysis applied to the glass-side ablation and delamination results in quite different conclusions. Although the lowest delamination threshold for the Ni(150 nm)/Au(5 nm) appears to be below the melting threshold of nickel at the Ni/Au interface, it remained large enough to heat the nickel at the Ni/Au interface to the melting temperature of gold (purple zone in Figure 6b). Therefore, similar to the previous case of the air-side ablation, we cannot rule out that the glass-side ablation/delamination evolves through the transient state of laser-melted gold. However, the nickel film in a Ni(150nm)/Au(5nm) bi-layer does not melt.

When comparing ablation and delamination thresholds to melting thresholds, it is evident that the underlying damage phenomena are mediated by the laser-induced melting at laser-excited surfaces/interfaces. At the same time, melting of the entire film at the delamination threshold can be ruled out, both for Ni/glass and Ni/Au/glass samples. Therefore, our study clearly demonstrates the possibility of the non-destructive nanofabrication of suspended metallic (multilayer) membranes via the femtosecond laser delamination mechanism.

## 5. Conclusions

In conclusion, we investigated ablation and delamination thresholds for Ni thin films and Ni/Au bilayers as a function of Ni thickness. The obtained data were compared to known experimental data and estimated melting threshold values. To estimate the melting thresholds, we performed intensity-dependent pump-probe measurements of transient reflectivity. Data analysis provided the value of the heat penetration depth (h = 25 nm) in nickel, a key parameter for threshold estimation.

Delamination thresholds were found to be lower than ablation thresholds for both Ni and Ni/Au, indicating that delamination is a less energy-consuming process for laser nanostructuring. The analysis based on the energy conservation law revealed that both ablation and delamination are driven by thermal effects, as reaching the melting temperature (of Ni and/or Au) at the laser-excited Ni/glass and Ni/Au/glass interface is necessary. Ablation and delamination thresholds for Ni/Au were lower than those for Ni. It follows from the very same energy conservation law that the absorbed laser fluence is not sufficient to melt the entire film, suggesting its significant fraction should remain in the solid state. This holds the potential for nondestructive, energy-efficient nanostructuring for the creation of high-quality acoustic resonators and hybrid nanostructures. Further insights are expected from single-shot time-resolved experiments.

## Figures and Tables

**Table 1:** The table of the coefficients *A* = (1 − *R* − *T)* for Ni and Ni/Au of different thicknesses.

**Ni**											
		20 nm		40 nm		60 nm		80 nm		100 nm	
*A*	Air Side	0.373		0.292		0.279		0.279		0.28	
	Glass Side	0.393		0.328		0.315		0.315		0.317	
**Ni/5 nm Au**											
		10 nm	30 nm		40 nm		70 nm		100 nm		150 nm
*A*	Air Side	0.421	0.309		0.29		0.28		0.28		0.28
	Glass Side	0.322	0.278		0.257		0.243		0.245		0.245

**Table 2:** Parameters for estimating the melting thresholds.

*ρ* (kg/m^3^) [44]	$c_{l}$ (J/kg × K) [44]	$T_{m}$ (K) [26]	$\Delta H_{m}$ (kJ/kg) [44]
8902	560	1728	293

## Data Availability

The data are available from the authors upon reasonable request.

## References

[1] Vorobyev A.Y., Guo C. (2011). Antireflection Effect of Femtosecond Laser-Induced Periodic Surface Structures on Silicon. Opt. Express.

[2] Xie X., Li Y., Wang G., Bai Z., Yu Y., Wang Y., Ding Y., Lu Z. (2022). Femtosecond Laser Processing Technology for Anti-Reflection Surfaces of Hard Materials. Micromachines.

[3] Sugioka K., Cheng Y. (2014). Ultrafast Lasers—Reliable Tools for Advanced Materials Processing. Light Sci. Appl..

[4] Kuchmizhak A., Vitrik O., Kulchin Y., Storozhenko D., Mayor A., Mirochnik A., Makarov S., Milichko V., Kudryashov S., Zhakhovsky V. (2016). Laser Printing of Resonant Plasmonic Nanovoids. Nanoscale.

[5] Ruffino F., Grimaldi M.G. (2019). Nanostructuration of Thin Metal Films by Pulsed Laser Irradiations: A Review. Nanomaterials.

[6] Li W., Thian E.S., Wang M., Wang Z., Ren L. (2021). Surface Design for Antibacterial Materials: From Fundamentals to Advanced Strategies. Adv. Sci..

[7] Lutey A.H.A., Gemini L., Romoli L., Lazzini G., Fuso F., Faucon M., Kling R. (2018). Towards Laser-Textured Antibacterial Surfaces. Sci. Rep..

[8] Stratakis E., Bonse J., Heitz J., Siegel J., Tsibidis G.D., Skoulas E., Papadopoulos A., Mimidis A., Joel A.-C., Comanns P. (2020). Laser Engineering of Biomimetic Surfaces. Mater. Sci. Eng. R Rep..

[9] Shchedrina N.N., Kudryashov S.I., Moskvin M.K., Odintsova G.V., Krylach I.V., Danilov P.A., Bondarenko A.G., Davydova E.A., Fokina M.I., Olekhnovich R.O. (2021). Elementary Autonomous Surface Microfluidic Devices Based on Laser-Fabricated Wetting Gradient Microtextures That Drive Directional Water Flows. Opt. Express.

[10] Chichkov B.N., Momma C., Nolte S., von Alvensleben F., Tünnermann A. (1996). Femtosecond, Picosecond and Nanosecond Laser Ablation of Solids. Appl. Phys. A Mater. Sci. Process..

[11] Bäuerle D. (2013). Laser Processing and Chemistry.

[12] Zhigilei L.V., Ivanov D.S. (2005). Channels of Energy Redistribution in Short-Pulse Laser Interactions with Metal Targets. Appl. Surf. Sci..

[13] von der Linde D., Sokolowski-Tinten K. (2000). The Physical Mechanisms of Short-Pulse Laser Ablation. Appl. Surf. Sci..

[14] Sokolowski-Tinten K., Bialkowski J., Cavalleri A., von der Linde D., Oparin A., Meyer-ter-Vehn J., Anisimov S.I. (1998). Transient States of Matter during Short Pulse Laser Ablation. Phys. Rev. Lett..

[15] Povarnitsyn M.E., Itina T.E., Sentis M., Khishchenko K.V., Levashov P.R. (2007). Material Decomposition Mechanisms in Femtosecond Laser Interactions with Metals. Phys. Rev. B Condens. Matter.

[16] Bonse J., Krüger J. (2022). Structuring of Thin Films by Ultrashort Laser Pulses. Appl. Phys. A Mater. Sci. Process..

[17] Fox J.A., Barr D.N. (1973). Laser-Induced Shock Effects in Plexiglas and 6061-T6 Aluminum. Appl. Phys. Lett..

[18] Salzmann D., Gilath I., Givon M., Bar-Noy T. (1989). Measurement of the Tensile Strength of Aluminium at a Strain Rate of 2 × 107s-1. J. Phys. D Appl. Phys..

[19] Tamura H., Kohama T., Kondo K., Yoshida M. (2001). Femtosecond-Laser-Induced Spallation in Aluminum. J. Appl. Phys..

[20] Korte F., Koch J., Chichkov B.N. (2004). Formation of Microbumps and Nanojets on Gold Targets by Femtosecond Laser Pulses. Appl. Phys. A Mater. Sci. Process..

[21] Domke M., Nobile L., Rapp S., Eiselen S., Sotrop J., Huber H.P., Schmidt M. (2014). Understanding Thin Film Laser Ablation: The Role of the Effective Penetration Depth and the Film Thickness. Phys. Procedia.

[22] Temnov V.V., Alekhin A., Samokhvalov A., Ivanov D.S., Lomonosov A., Vavassori P., Modin E., Veiko V.P. (2020). Nondestructive Femtosecond Laser Lithography of Ni Nanocavities by Controlled Thermo-Mechanical Spallation at the Nanoscale. Nano Lett..

[23] Kim J.-W., Bigot J.-Y. (2017). Magnetization Precession Induced by Picosecond Acoustic Pulses in a Freestanding Film Acting as an Acoustic Cavity. Phys. Rev. B Condens. Matter.

[24] Ghita A., Mocioi T.-G., Lomonosov A.M., Kim J., Kovalenko O., Vavassori P., Temnov V.V. (2023). Anatomy of Ultrafast Quantitative Magnetoacoustics in Freestanding Nickel Thin Films. Phys. Rev. B Condens. Matter.

[25] Xiao S., Schöps B., Ostendorf A. (2012). Selective Ablation of Thin Films by Ultrashort Laser Pulses. Phys. Procedia.

[26] Wellershoff S.-S., Hohlfeld J., Güdde J., Matthias E. (1999). The Role of Electron–phonon Coupling in Femtosecond Laser Damage of Metals. Appl. Phys. A Mater. Sci. Process..

[27] Velli M.-C., Maragkaki S., Vlahou M., Tsibidis G.D., Stratakis E. (2024). Investigation of the Role of Pulse Duration and Film Thickness on the Damage Threshold of Metal Thin Films Irradiated with Ultrashort Laser Pulses. Appl. Surf. Sci..

[28] Mishra K., Rowan-Robinson R.M., Ciuciulkaite A., Davies C.S., Dmitriev A., Kapaklis V., Kimel A.V., Kirilyuk A. (2022). Ultrafast Demagnetization Control in Magnetophotonic Surface Crystals. Nano Lett..

[29] Frolov A.Y., Shcherbakov M.R., Fedyanin A.A. (2020). Dark Mode Enhancing Magneto-Optical Kerr Effect in Multilayer Magnetoplasmonic Crystals. Phys. Rev. B Condens. Matter.

[30] Manke K.J., Maznev A.A., Klieber C., Shalagatskyi V., Temnov V.V., Makarov D., Baek S.-H., Eom C.-B., Nelson K.A. (2013). Detection of Shorter-than-Skin-Depth Acoustic Pulses in a Metal Film via Transient Reflectivity. Appl. Phys. Lett..

[31] Shalagatskyi V. (2015). Ultrafast Acoustics in Hybrid and Magnetic Structures. Ph.D. Dissertation.

[32] Zhang D.-L., Zhu J., Qu T., Lattery D.M., Victora R.H., Wang X., Wang J.-P. (2020). High-Frequency Magnetoacoustic Resonance through Strain-Spin Coupling in Perpendicular Magnetic Multilayers. Sci. Adv..

[33] Thevenard L., Peronne E., Gourdon C., Testelin C., Cubukcu M., Charron E., Vincent S., Lemaître A., Perrin B. (2010). Effect of Picosecond Strain Pulses on Thin Layers of the Ferromagnetic Semiconductor (Ga,Mn)(As,P). Phys. Rev. B Condens. Matter.

[34] Temnov V.V., Klieber C., Nelson K.A., Thomay T., Knittel V., Leitenstorfer A., Makarov D., Albrecht M., Bratschitsch R. (2013). Femtosecond Nonlinear Ultrasonics in Gold Probed with Ultrashort Surface Plasmons. Nat. Commun..

[35] Suslova A., Hassanein A. (2017). Femtosecond Laser Absorption, Heat Propagation, and Damage Threshold Analysis for Au Coating on Metallic Substrates. Appl. Surf. Sci..

[36] Ray A. (2023). Two-Temperature Model for Ultrafast Melting of Au-Based Bimetallic Films Interacting with Single-Pulse Femtosecond Laser: Theoretical Study of Damage Threshold. Phys. Rev. B Condens. Matter.

[37] Kudryashov S.I., Gakovic B., Danilov P.A., Petrovic S.M., Milovanovic D., Rudenko A.A., Ionin A.A. (2018). Single-Shot Selective Femtosecond Laser Ablation of Multi-Layered Ti/Al and Ni/Ti Films: “Cascaded” Heat Conduction and Interfacial Thermal Effects. Appl. Phys. Lett..

[38] Romashevskiy S.A., Khokhlov V.A., Ashitkov S.I., Zhakhovsky V.V., Inogamov N.A., Komarov P.S., Parshikov A.N., Petrov Y.V., Struleva E.V., Tsygankov P.A. (2021). Femtosecond Laser Irradiation of a Multilayer Metal–Metal Nanostructure. JETP Lett..

[39] Lian Y., Jiang L., Sun J., Tao W., Chen Z., Lin G., Ning Z., Ye M. (2023). Ultrafast Dynamics and Ablation Mechanism in Femtosecond Laser Irradiated Au/Ti Bilayer Systems. Nanophotonics.

[40] Vernik U., Lomonosov A.M., Vlasov V.S., Kotov L.N., Kuzmin D.A., Bychkov I.V., Vavassori P., Temnov V.V. (2022). Resonant Phonon-Magnon Interactions in Freestanding Metal-Ferromagnet Multilayer Structures. Phys. Rev. B Condens. Matter.

[41] Liu J.M. (1982). Simple Technique for Measurements of Pulsed Gaussian-Beam Spot Sizes. Opt. Lett..

[42] Knittl Z. (1976). Optics of Thin Films: An Optical Multilayer Theory.

[43] Yeh P., Hendry M. (1990). Optical Waves in Layered Media. Phys. Today.

[44] Siegel J., Matthias E., Ettrich K., Welsch E. (1997). UV-Laser Ablation of Ductile and Brittle Metal Films. Appl. Phys. A Mater. Sci. Process..

[45] Corkum P.B., Brunel F., Sherman N.K., Srinivasan-Rao T. (1988). Thermal Response of Metals to Ultrashort-Pulse Laser Excitation. Phys. Rev. Lett..

[46] Thomsen C., Grahn H.T., Maris H.J., Tauc J. (1986). Surface Generation and Detection of Phonons by Picosecond Light Pulses. Phys. Rev. B Condens. Matter.

[47] Devos A., Lerouge C. (2001). Evidence of Laser-Wavelength Effect in Picosecond Ultrasonics: Possible Connection with Interband Transitions. Phys. Rev. Lett..

[48] Saito T., Matsuda O., Wright O.B. (2003). Picosecond Acoustic Phonon Pulse Generation in Nickel and Chromium. Phys. Rev. B Condens. Matter.

[49] Medvedev N., Milov I. (2020). Electron-Phonon Coupling in Metals at High Electronic Temperatures. Phys. Rev. B Condens. Matter.

[50] Lin Z., Zhigilei L.V., Celli V. (2008). Electron-Phonon Coupling and Electron Heat Capacity of Metals under Conditions of Strong Electron-Phonon Nonequilibrium. Phys. Rev. B Condens. Matter.

